# 

**Published:** 1879-03

**Authors:** 


A Monograph on the Treatment of Diphtheria. By William C. Reiter, A. M., M. D.; Philadelphia, J. B. Lippincott & Co., pp. 47.

An Introduction to Pathology and Morbid Anatomy. By T. Henry Green, M. D., London. Philadelphia: Henry C. Lea: Buffalo, T. H. Butler, pp. 323.

A Tabular Handbook of Auscultation and Percussion. By Herbert C. Clapp, A. M , M. D ; Boston, Houghton, Osgood & Co.; Buffalo, Peter Paul & Brother, pp 97, price $1.50.

Medical Chemistry, Including the Outlines of Organic and Physiological Chemistry. By C. Gilbert Wheeler, Prof. of Chemistry in the University of Chicago; Philadelphia, Lindsay & Blakiston; Chicago, S. J. Wheeler; Buffalo, Theo. H. Butler, pp. 409, price $3.00.

A Manual for the Practice of Surgery. By Thomas Bryant, F. R. C. S., with six hundred and seventy-two Illustrations; Philadelphia, Henry C. Lea; Buffalo, Theo. H. Butler, pp. 932.

Index to Original Communications in the Medical Journals of the United States and Canada, for 1877. Compiled by Wm. D. Chapin, New York, pp. 48, price $1.00.

Excerpta from the Annual Report to the Board of Health for 1878 By Joseph Holt, M. D., Sanitary Inspector of the Fourth District New Orleans.

Diphtheria and its Treatment. By Chas. H. S. Davis, M. D., Meriden, Conn.

Electricity in its Relations to Medicine and Surgery. By A. D. Rockwell, M. D.

First Annual Report of the Board of Health of the city of Buffalo.

A Treatise on the Horse and his Diseases. By B. J. Kendall, M. D.

(4 c/r)

INDEXED

### THE BUFFALO MEDICAL AND SURGICAL JOURNAL.

MEDICAL AND SURGICAL JOURNAL.

PUBLISHED MONTHLY.—Containing Original Articles, Reports of Medical Societies and Hospitals, Editorial, Reviews, Correspondence, Army News, etc., etc; including the usual variety of Medical Periodical Publications. Specimen copies sent on application to BUFFALO MEDICAL AND SURGICAL JOURNAL, J. F. MINER, M. D., 978 Main Street, BUFFALO, N. Y.

TERMS OF SUBSCRIPTION, $3 00 PER YEAR, IN ADVANCE,—All communications for publication should be sent before the fifteenth (15) of each month, or they will of necessity lie over until the next number. No change can be made in advertisements after the twenty-fifth.

Correspondence and original articles are solicited. Publication is no evidence that the views of the author are endorsed by the Journal.

